# Sports-team participation and risk behaviors among US high school students: behavior-specific and sex-heterogeneous associations in the national YRBS, 2011–2023

**DOI:** 10.3389/fpubh.2026.1975621

**Published:** 2026-10-07

**Authors:** Fa-liang Sun, Qing Liu

**Affiliations:** School of Physical Education and Sports Science, South China Normal University, Guangzhou, China

**Keywords:** adolescent health, organized sport, risk behavior, sex differences, youth sport

## Abstract

Sports-team participation is often regarded as a favorable adolescent context, but whether its associations are consistent across distinct risk behaviors and across sex remains unresolved. We conducted a repeated cross-sectional analysis of seven U. S. National Youth Risk Behavior Survey cycles (2011–2023; 110,409 records) using survey-weighted regression and marginal standardization. Participation in one or more sports teams was associated with higher adjusted prevalence of alcohol use (PD, +4.46 percentage points; 95% CI, +3.38 to +5.55) and physical fighting (+2.53; 95% CI, +1.62 to +3.43), but lower prevalence of cigarette smoking (−3.46; 95% CI, −4.10 to −2.82) and marijuana use (−1.49; 95% CI, −2.33 to −0.66); all Holm-adjusted *p* < 0.001. Sex heterogeneity was observed for all four outcomes (all interaction *p* < 0.001): positive associations with fighting and alcohol were more pronounced among males, whereas inverse associations with cigarette smoking and marijuana use were more evident among females. The overall behavioral pattern was retained in participation-level and prespecified sensitivity analyses, although alcohol showed evidence of survey-cycle heterogeneity. Sports-team participation therefore corresponded to a behavior-specific and sex-heterogeneous, rather than uniformly lower- or higher-risk, behavioral profile. These findings may inform prevention science and intervention development in U. S. high school settings by supporting behavior-specific, context-sensitive strategies that address the social conditions surrounding sports participation rather than treating participation itself as uniformly protective or harmful.

## 1 Introduction

Organized sports are widely regarded as a favorable context for adolescent development. Given the widespread participation of adolescents in organized sport and the continuing prevalence of multiple health-risk behaviors, understanding whether sports participation corresponds to consistent or divergent behavioral patterns remains an important public health question. In the 2023 National Youth Risk Behavior Survey (YRBS), 51.9% of U. S. high school students reported playing on at least one school or community sports team during the preceding 12 months. In the same survey, 19.2% reported physical fighting during the preceding 12 months, while 22.1, 3.5, and 17.0% reported alcohol use, cigarette smoking, and marijuana use, respectively, during the preceding 30 days (1). However, sports participation is not necessarily associated with uniformly favorable behavioral outcomes. Physical fighting, alcohol use, cigarette smoking, and marijuana use represent distinct behavioral domains, each with its own social meaning, opportunity structure, and developmental relevance. Problem Behavior Theory provides the overarching framework for this study by conceptualizing adolescent behavior as arising from interactions among individual characteristics, perceived social environments, and broader behavioral systems, rather than from any single activity or exposure (2). Within this framework, the same sporting context may be associated with different behavioral patterns because peer norms, behavioral opportunities, and social constraints may operate differently across outcomes. Sports participants may therefore exhibit a combination of characteristics associated with both positive and negative behaviors, rather than a uniformly “healthy” or “high-risk” profile. Evidence synthesized across multiple reviews similarly indicates that organized sports can be associated with both favorable and unfavorable health-related outcomes (3). Sports participation may thus be better understood as a social context whose behavioral correlates vary by outcome, rather than as an inherently protective or harmful exposure.

Social Cognitive Theory complements this overarching framework by providing an additional perspective for interpreting how self-efficacy, outcome expectations, and perceived social environments may shape behavioral choices within social contexts (4). In particular, differences in anticipated consequences, peer approval, and self-regulatory processes may help explain why participation in the same sporting environment may correspond to different behavioral outcomes. Thus, the two theories serve complementary roles: Problem Behavior Theory organizes the broader individual and social domains associated with adolescent risk behavior, whereas Social Cognitive Theory provides a perspective for understanding behavioral regulation processes within those domains. This theoretical integration is consistent with Luis et al.’s review, which positioned an overarching ecological framework as a means of integrating developmental, motivational, and psychosocial perspectives when interpreting complex adolescent behaviors (5). These perspectives guide interpretation of the present findings but do not represent mechanisms directly tested in this study.

Previous research has documented such outcome-specific differences, although the patterns have not been consistent across studies. Longitudinal studies and systematic reviews have generally reported a relatively consistent positive association between sports participation and alcohol use, whereas associations with cigarette smoking, marijuana use, and other illicit drug use tend to be inverse or more heterogeneous (6–8). Individual longitudinal studies have further found more rapid increases in problematic alcohol use among some sports participants (9), higher rates of alcohol initiation but lower rates of marijuana initiation among team-sport participants (10), and a greater subsequent risk of alcohol intoxication alongside lower trajectories of tobacco and marijuana use in certain sporting contexts (11). These associations, however, are not universal. More recent longitudinal evidence has found little systematic correspondence between changes in sports participation and changes in alcohol use (12). Findings for violence-related behaviors are similarly mixed. Meta-analytic evidence suggests no overall association between sports participation and juvenile delinquency (13), while research on peer contexts has identified no consistent relationship between sports participation and violent offending (14). By contrast, analyses of specific sports indicate that serious fighting may be concentrated in particular contact sports and peer-network environments (15). Taken together, the available evidence points to heterogeneity across behavioral outcomes and sporting contexts rather than a single, uniform effect of sports participation.

Sex may represent an additional dimension of this heterogeneity, yet previous research has not established a generalizable pattern of differences between male and female adolescents. Canadian data indicate that binge drinking is common among team-sport participants of both sexes, whereas female participants report lower levels of cigarette and marijuana use; these associations also vary according to individuals’ physical involvement and social integration within team-sport culture (16). Early U. S. studies using YRBS data likewise identified sex-specific patterns of substance use among sports participants (17). Longitudinal studies have reported both sex-specific associations (18, 19) and an absence of sex moderation for certain alcohol-related outcomes (9). These inconsistencies may reflect differences in sport type, competitive environment, peer relationships, study population, developmental stage, or measurement approach rather than a stable sex effect. Related population-based studies further indicate that moderation may vary across exposure–outcome pathways. In an analysis of the 2011–2023 YRBS, Luis reported that sex moderated direct associations between school bullying and suicidal behaviors, whereas physical activity moderated indirect associations through depressive symptoms (20). In U. S. adults with disabilities, physical activity moderated the direct association between body mass index and depressive disorders, but did not significantly modify the indirect association through healthcare access (21). These findings highlight the importance of examining conditional associations while also emphasizing that differences in exposures, outcomes, and populations limit direct extrapolation to adolescent sports-team participation. It therefore remains unclear whether outcome-specific and sex-specific associations can be observed simultaneously when multiple outcomes are examined within the same nationally representative population using consistent exposure definitions and a common analytical framework.

To address this gap, the present study used data from seven cycles of the U. S. National YRBS conducted between 2011 and 2023. Applying a consistent analytical framework that accounted for the complex survey design and used marginal standardization, we examined associations between participation on sports teams during the preceding 12 months and four primary outcomes: physical fighting, current alcohol use, current cigarette smoking, and current marijuana use. We further characterized outcome distributions across reported levels of sports-team participation; assessed heterogeneity by sex and survey year; examined two related secondary outcomes; and conducted sensitivity analyses based on survey cycles and questionnaire wording. Given the heterogeneity of the existing evidence, we did not hypothesize a common direction of association across all outcomes. Instead, our central question was whether sports-team participation corresponds to a shared behavioral risk profile or whether its associations are better characterized as outcome-specific and heterogeneous by sex.

## 2 Materials and methods

### 2.1 Data source, design, and study population

This observational repeated cross-sectional study used National Youth Risk Behavior Survey (YRBS) data from 2011, 2013, 2015, 2017, 2019, 2021, and 2023. The National YRBS uses a weighted three-stage cluster sampling design to obtain nationally representative samples of students in grades 9–12 attending public and private schools in the United States (22, 23). Each survey cycle samples a new group of schools and students; observations therefore do not represent longitudinal follow-up of the same individuals.

Across the seven eligible cycles, 110,409 records were available before outcome-specific complete-case restriction. The full complex-survey design was specified before analytic domains were defined, and each outcome was subsequently analyzed within its defined analytic domain rather than by deleting observations before design construction. Analyses followed the prespecified analytic plan.

### 2.2 Measures

**Exposure.** The primary exposure was past-year participation on one or more school or community sports teams vs. no teams. For the primary analysis, sports-team participation was coded as 0 for no team participation and 1 for participation in one or more teams. Secondary analyses represented reported team participation as 0, 1, 2, or ≥3 teams, with categorical contrasts relative to 0 teams and a separately estimated ordinal trend. For the ordinal trend analysis, these categories were coded numerically as 0, 1, 2, and 3, with 3 representing three or more teams. Team count does not measure sport type, contact classification, training volume, competitive level, season, team culture, or duration of participation and was therefore interpreted as a participation indicator rather than a biological or behavioral dose.

**Outcomes.** The four primary outcomes were physical fighting at least once in the past 12 months, current alcohol use on at least 1 day in the past 30 days, current cigarette smoking on at least 1 day in the past 30 days, and current marijuana use at least once in the past 30 days. Secondary outcomes were physical fighting on school property at least once in the past 12 months and binge drinking during 2017–2023. Binge drinking was analyzed only for the 2017, 2019, 2021, and 2023 cycles because the harmonized indicator was structurally unavailable in earlier cycles. Outcome definitions were harmonized across eligible cycles; a planned marijuana analysis restricted the sample to 2011–2019 to address the questionnaire-wording concern. The current-cigarette-smoking outcome referred specifically to conventional combustible cigarettes; e-cigarettes and other electronic nicotine delivery systems were not included in this outcome. All CDC-derived binary outcome indicators were recoded as 1 when the specified behavior was present and 0 when it was absent, with missing or unclassifiable responses retained as missing.

**Covariates.** All adjusted models included sex, grade, seven-category race/ethnicity, and survey year. The available YRBS sex variable was binary; analyses are therefore described as sex-specific rather than gender-specific. The seven-category race/ethnicity specification was retained because the predefined conditions for using the alternative four-category coding were not met. For regression analyses, categorical covariates were entered using prespecified reference categories. Female sex, ninth grade, White race/ethnicity, and the 2011 survey cycle were the reference categories in the all-period models. Survey year was modeled categorically rather than continuously to distinguish independently sampled survey cycles.

Exposure, outcome, sex, grade, and race/ethnicity measures were derived from students’ self-reported questionnaire responses, whereas survey year was defined by the survey cycle. The potential implications of the shared self-report measurement source are considered in Section 4.4. Detailed analytical coding, event definitions, and reference categories for all study variables are provided in Supplementary Table S1.

### 2.3 Statistical analysis

Analyses followed four components defined in the analysis plan: estimation of the primary adjusted associations, evaluation of patterns across reported levels of sports-team participation, assessment of heterogeneity by sex and survey year, and multiplicity control and sensitivity analyses addressing alternative relative-association estimation, missingness, and questionnaire differences.

All analyses accounted for the complex sampling design of the National YRBS. Original survey weights were retained, and strata and primary sampling units were nested within survey year to distinguish sampling units across independently conducted survey cycles. The survey design was specified on the pooled dataset before outcome-specific eligibility restrictions were applied; analyses were subsequently conducted within predefined analytic domains so that the underlying sampling structure was preserved. Single-primary-sampling-unit strata, including those arising within analytic domains, were handled using the corresponding adjustment implemented in the R survey package.

For each primary and secondary outcome, the adjusted association with sports-team participation was first estimated using a survey-weighted logistic regression model:


$$\log[\frac{p(Y_{i}=1|S_{i}X_{i})}{1-p(Y_{i}=1|S_{i}X_{i})}]=\beta_{0}+\beta_{1}S_{i}+\beta^{T}X_{i}$$ (1)

where *Y_i_* denotes the binary risk-behavior outcome for student *i*; *S_i_* = 1 indicates participation in one or more sports teams and *S_i_* = 0 indicates no team participation; and *X_i_* contains the covariates sex, grade, seven-category race/ethnicity, and survey year, with survey year treated categorically. The coefficient *β_1_* represents the adjusted conditional association on the log-odds scale. Because conditional odds ratios were not the primary estimands of interest, interpretation focused on marginally standardized prevalence measures.

For marginal standardization, the fitted model was used to predict each student’s probability of the outcome under each exposure level s∈{0,1}, while retaining that student’s observed covariate values. These predicted probabilities were averaged over the survey-weighted covariate distribution of the relevant outcome-specific analytic domain:


$${\hat{p}}_{s}=\frac{\sum_{i\in D}w_{i}{\hat{p}}_{i}(s)}{\sum_{i\in D}w_{i}}$$ (2)

where *D* denotes the outcome-specific analytic domain, *w_i_* is the original survey weight, and ${\hat{p}}_{i}(s)$ is the model-predicted probability for student *i* after setting sports-team participation to *s*. Accordingly, ${\hat{p}}_{0}$ and ${\hat{p}}_{1}$ denote marginally standardized probabilities on the 0–1 scale. The primary absolute association measure was the adjusted prevalence difference (PD),


$$\mathrm{PD}=100(\hat{P_{1}}-\hat{P_{0}})$$ (3)

reported in percentage points. The standardized prevalence ratio (PR) was used as a complementary relative association measure:


$$\mathrm{PR}=\frac{\hat{P_{1}}}{\hat{P_{0}}}$$ (4)

Design-based 95% confidence intervals were calculated for marginal prevalences and their corresponding contrasts. PD was designated as the primary absolute association measure because it expresses the adjusted difference on an absolute prevalence scale, whereas the standardized PR provides complementary information about the relative magnitude of the association.

**Participation-level analyses.** Sports-team participation was also examined across four reported team-count categories (0, 1, 2, and ≥3 teams). Categorical models treated 0 teams as the reference category and were used to estimate marginal prevalences across participation levels. A separate ordinal specification evaluated the planned linear trend across the four ordered categories. Because team count does not quantify training volume, competitive exposure, sport intensity, or duration of participation, these estimates were interpreted as participation-level patterns rather than dose–response effects.

**Heterogeneity analyses.** Interactions between sports-team participation and sex and between sports-team participation and survey year were added to the corresponding survey-weighted logistic models. Overall evidence of heterogeneity was assessed using joint design-based tests of the interaction terms rather than by comparing whether subgroup-specific estimates were individually statistically significant. Where relevant, sex- and survey-year-specific adjusted prevalences, PDs, and standardized PRs were obtained by marginal standardization of the interaction models.

**Multiplicity and relative-association sensitivity analyses.** Multiplicity correction was restricted to the four primary PD comparisons specified in advance. Their *p* values were adjusted using the Holm procedure to control the family-wise error rate. As a sensitivity analysis for the relative association measure, survey-weighted modified Poisson models with a log link were fitted for the primary outcomes. These estimates were used to assess whether the relative association pattern was consistent with the standardized PRs derived from the logistic models and did not replace the marginal-standardization framework used for the primary analyses.

**Missing data and cycle/wording sensitivity analyses.** Missing data were handled using outcome-specific complete-case analytic domains defined in the analysis plan rather than a single complete-case sample shared across all outcomes. Sports-team participation was more frequently missing or unclassifiable in later survey cycles, and the available survey documentation did not allow this pattern to be attributed definitively to questionnaire structure, item nonresponse, administration, or data-processing mechanisms. Because the source of exposure missingness was uncertain and potentially structural, missing sports-team participation values were not imputed. To examine whether the primary associations were disproportionately dependent on the later survey cycles, analyses were repeated after excluding 2021, excluding 2023, and excluding both cycles. Current marijuana use was additionally re-estimated using 2011–2019 data to address the questionnaire-wording concern. These checks assessed the stability of the direction of the primary associations under alternative cycle and wording restrictions; they were not used to assume that the underlying missingness process had been resolved.

All analyses were conducted in R version 4.6.1 using the survey package (version 4.5).

## 3 Results

### 3.1 Sample and analytic population

Across the seven National YRBS cycles, the combined dataset contained 110,409 records. In the survey-weighted sample, 48.6% were female and 50.6% were male, with 0.8% missing or unclassifiable sex information. Sports-team participation was reported as no teams by 39.9% of the sample and one or more teams by 48.0%; 12.1% had missing or unclassifiable exposure information, which was more concentrated in later survey cycles.

Because analyses were conducted within prespecified outcome-specific complete-case domains, analytic sample sizes differed across the four primary outcomes, ranging from 76,909 for current alcohol use to 82,362 for current marijuana use. The primary analyses therefore did not use a single common complete-case sample. Characteristics of the combined sample are presented in Table 1.

**Table 1:** Characteristics of the combined national YRBS sample, 2011–2023.

Characteristic	Category	Survey-weighted %
Sex	Female	48.6
	Male	50.6
	Missing or not classifiable	0.8
Grade	9th grade	26.8
	10th grade	25.5
	11th grade	23.9
	12th grade	23.0
	Missing or not classifiable	0.9
Race/ethnicity	White	51.5
	American Indian or Alaska Native	0.6
	Asian	3.9
	Black or African American	13.0
	Hispanic or Latino	23.3
	Native Hawaiian or other Pacific Islander	0.6
	Multiple race, non-Hispanic	4.9
	Missing or not classifiable	2.2
Sports-team participation	0 teams	39.9
	≥1 team	48.0
	Missing or not classifiable	12.1

The combined dataset contained 110,409 unweighted records. Percentages are survey-weighted and may not sum to 100% because of rounding. Missing or unclassifiable categories are retained to describe the composition of the analysis-ready sample.

### 3.2 Behavior-specific associations and participation-level patterns

Sports-team participation was associated with contrasting patterns across the four primary risk behaviors (Table 2; Figure 1). On the absolute scale, current alcohol use showed the largest positive association with participation in one or more teams (PD, +4.46 percentage points; 95% CI, +3.38 to +5.55), followed by physical fighting (+2.53; 95% CI, +1.62 to +3.43). In contrast, current cigarette smoking showed the largest inverse association (−3.46; 95% CI, −4.10 to −2.82), while the inverse association with marijuana use was smaller (−1.49; 95% CI, −2.33 to −0.66). All four primary PD comparisons remained supported after Holm adjustment (*p* < 0.001).

**Table 2:** Adjusted associations between sports-team participation and primary risk behaviors.

Outcome	Analytic N	Adjusted prevalence, 0 teams, %	Adjusted prevalence, ≥1 team, %	PD, percentage points (95% CI)	Standardized PR (95% CI)	Holm-adjusted *p*
Physical fighting	80,325	21.6	24.1	+2.53 (+1.62 to +3.43)	1.12 (1.07–1.16)	<0.001
Current alcohol use	76,909	27.7	32.1	+4.46 (+3.38 to +5.55)	1.16 (1.12–1.20)	<0.001
Current cigarette smoking	80,342	11.4	7.9	−3.46 (−4.10 to −2.82)	0.70 (0.65–0.74)	<0.001
Current marijuana use	82,362	21.0	19.5	−1.49 (−2.33 to −0.66)	0.93 (0.89–0.97)	<0.001

Analytic N denotes the unweighted outcome-specific analytic sample. Adjusted prevalences were obtained by marginal standardization from survey-weighted logistic regression models. PD denotes the adjusted prevalence difference for participation in one or more sports teams vs. no teams, expressed in percentage points; PR denotes the standardized prevalence ratio. Models adjusted for sex, grade, seven-category race/ethnicity, and survey year and accounted for the complex YRBS sampling design. Holm-adjusted p values correspond to the four primary PD comparisons specified in advance.

**Figure 1:**
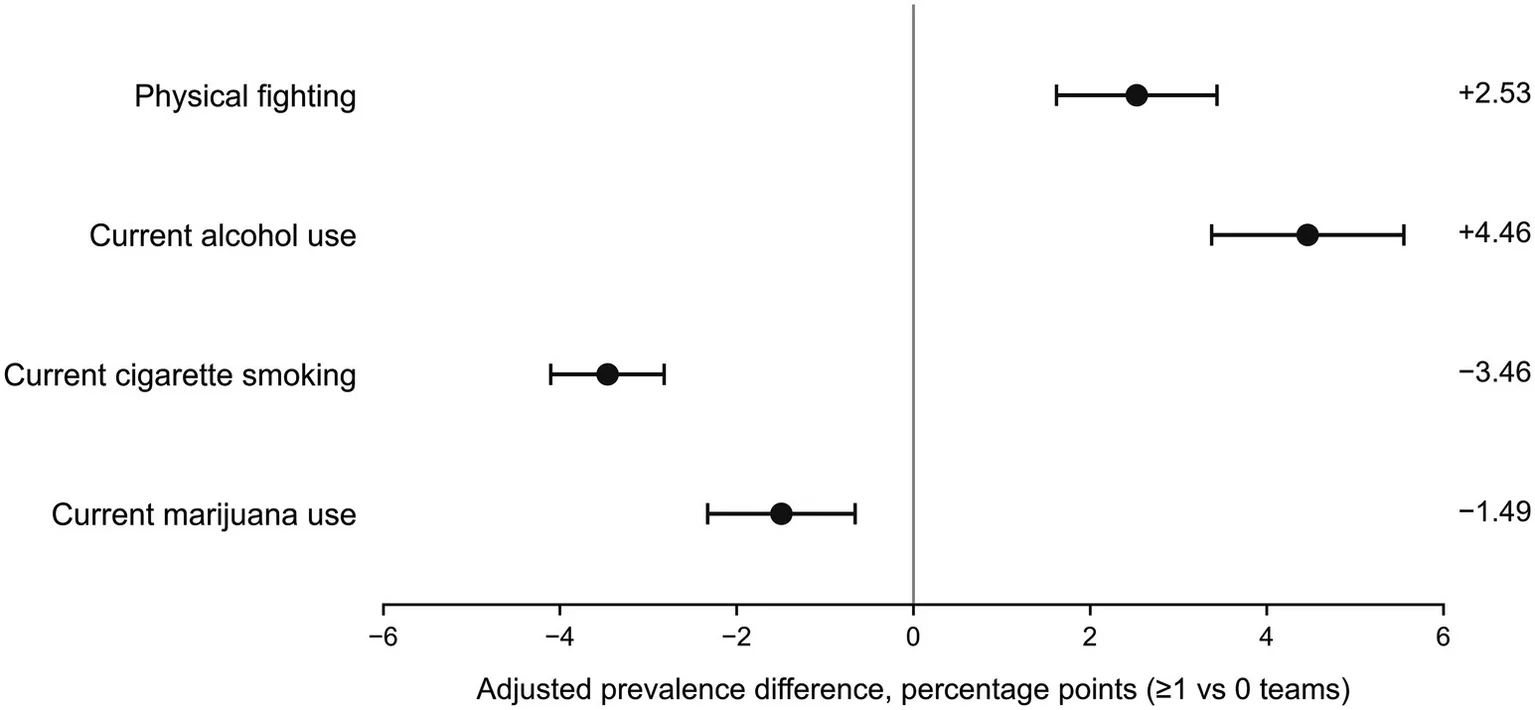
Adjusted prevalence differences in primary risk behaviors by sports-team participation. Points show adjusted prevalence differences (PDs; ≥1 vs. 0 teams), and horizontal error bars show design-based 95% confidence intervals. Estimates were obtained by marginal standardization from survey-weighted logistic regression models adjusted for sex, grade, seven-category race/ethnicity, and survey year while accounting for the complex National YRBS sampling design. The vertical reference line denotes no prevalence difference (PD = 0); positive values indicate higher adjusted prevalence among students reporting participation in ≥1 sports team.

Associations across reported team-count categories reproduced the same behavioral split but differed in shape across outcomes (Figure 2). Physical fighting increased progressively across participation levels. Alcohol use increased most sharply between no participation and one team and then rose more gradually. Cigarette smoking declined through the one- and two-team categories but changed little thereafter, with a slight increase among students reporting three or more teams. Marijuana use showed a comparatively gradual decline across participation levels.

**Figure 2:**
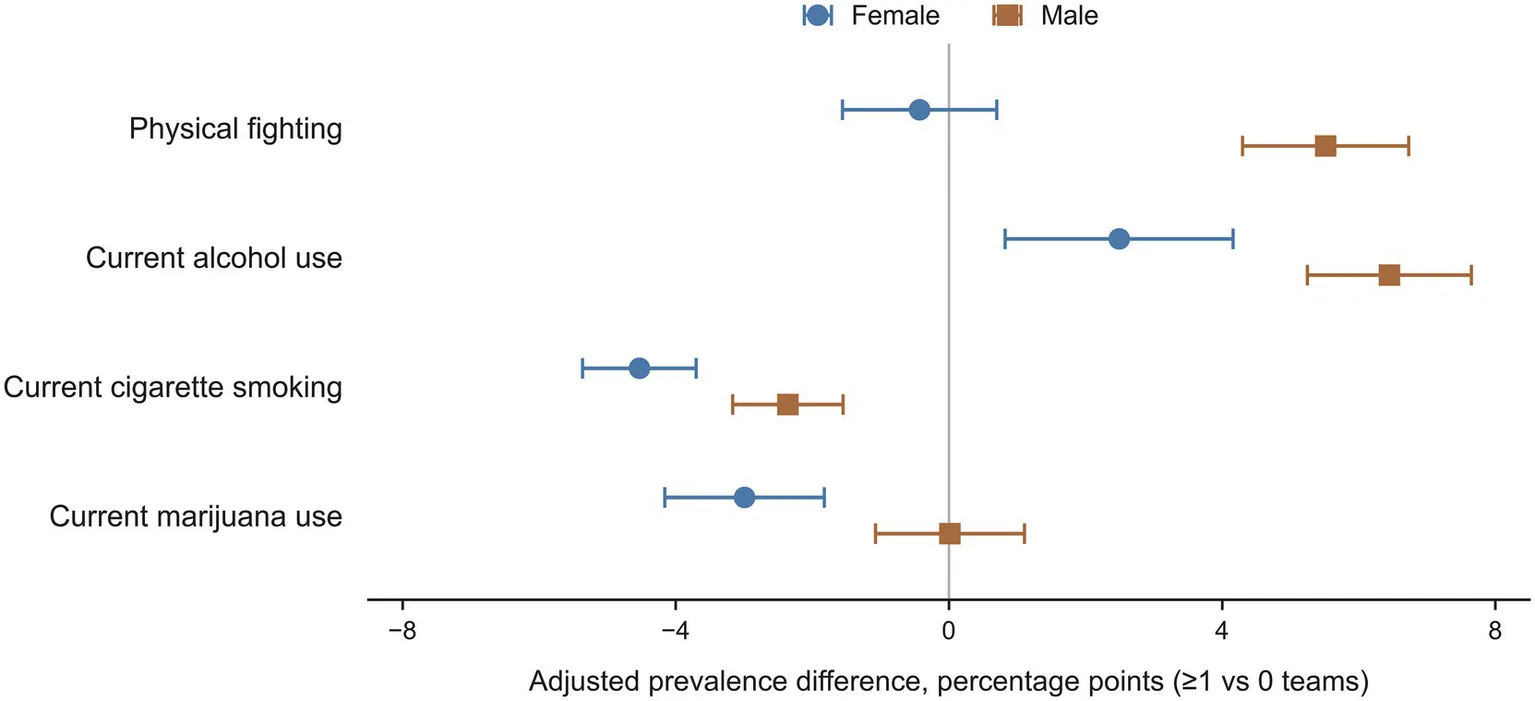
Adjusted prevalence of primary risk behaviors across reported numbers of sports teams. Points and error bars show adjusted prevalences obtained by marginal standardization and their design-based 95% confidence intervals for students reporting 0, 1, 2, and ≥3 sports teams. Estimates were derived from survey-weighted logistic regression models adjusted for sex, grade, seven-category race/ethnicity, and survey year while accounting for the complex National YRBS sampling design. Lines connect category-specific estimates for visual presentation and do not imply a linear or dose–response relationship. Y-axis ranges differ across panels and are intended to display within-outcome patterns rather than facilitate direct comparison of slopes across outcomes.

The corresponding ordinal trend estimates were positive for physical fighting (OR per category, 1.09; 95% CI, 1.06–1.12) and alcohol use (OR, 1.11; 95% CI, 1.07–1.14), and inverse for cigarette smoking (OR, 0.82; 95% CI, 0.79–0.85) and marijuana use (OR, 0.95; 95% CI, 0.92–0.98; all *p* < 0.001). Thus, binary and participation-level analyses converged on the same broad behavior-specific pattern. The nonmonotonic cigarette-smoking pattern, in particular, indicates that reported team count should not be interpreted uniformly as a dose–response measure across outcomes.

### 3.3 Sex heterogeneity in behavior-specific associations

The interaction between sports-team participation and sex was supported for all four primary outcomes (all joint *p* < 0.001; Table 3; Figure 3). Two forms of heterogeneity were apparent. For physical fighting and marijuana use, the pooled associations masked a near-null estimate in one sex stratum. The positive fighting association was concentrated among males (PD, +5.51 percentage points; 95% CI, +4.30 to +6.73), whereas the female estimate was near null (−0.43; 95% CI, −1.56 to +0.70). Conversely, marijuana use was inversely associated with participation among females (PD, −2.99; 95% CI, −4.16 to −1.83), while the male estimate was essentially null (+0.01; 95% CI, −1.08 to +1.10).

**Table 3:** Sex-specific adjusted associations between sports-team participation and primary risk behaviors.

Outcome	Female PD, percentage points (95% CI)	Female standardized PR (95% CI)	Male PD, percentage points (95% CI)	Male standardized PR (95% CI)	Joint interaction *p*
Physical fighting	−0.43 (−1.56 to +0.70)	0.97 (0.91–1.04)	+5.51 (+4.30 to +6.73)	1.21 (1.16–1.27)	<0.001
Current alcohol use	+2.49 (+0.82 to +4.16)	1.08 (1.03–1.14)	+6.45 (+5.25 to +7.65)	1.26 (1.21–1.32)	<0.001
Current cigarette smoking	−4.53 (−5.36 to −3.70)	0.59 (0.53–0.64)	−2.36 (−3.17 to −1.55)	0.80 (0.74–0.86)	<0.001
Current marijuana use	−2.99 (−4.16 to −1.83)	0.86 (0.81–0.91)	+0.01 (−1.08 to +1.10)	1.00 (0.95–1.05)	<0.001

PD denotes the adjusted prevalence difference for participation in one or more sports teams vs. no teams; PR denotes the standardized prevalence ratio. Estimates were derived from survey-weighted interaction models and marginal standardization. Models included the main effects of sports-team participation and sex, their interaction, and adjustment for grade, seven-category race/ethnicity, and survey year while accounting for the complex YRBS sampling design. Joint interaction p values test whether the association between sports-team participation and each outcome differed by sex.

**Figure 3:**
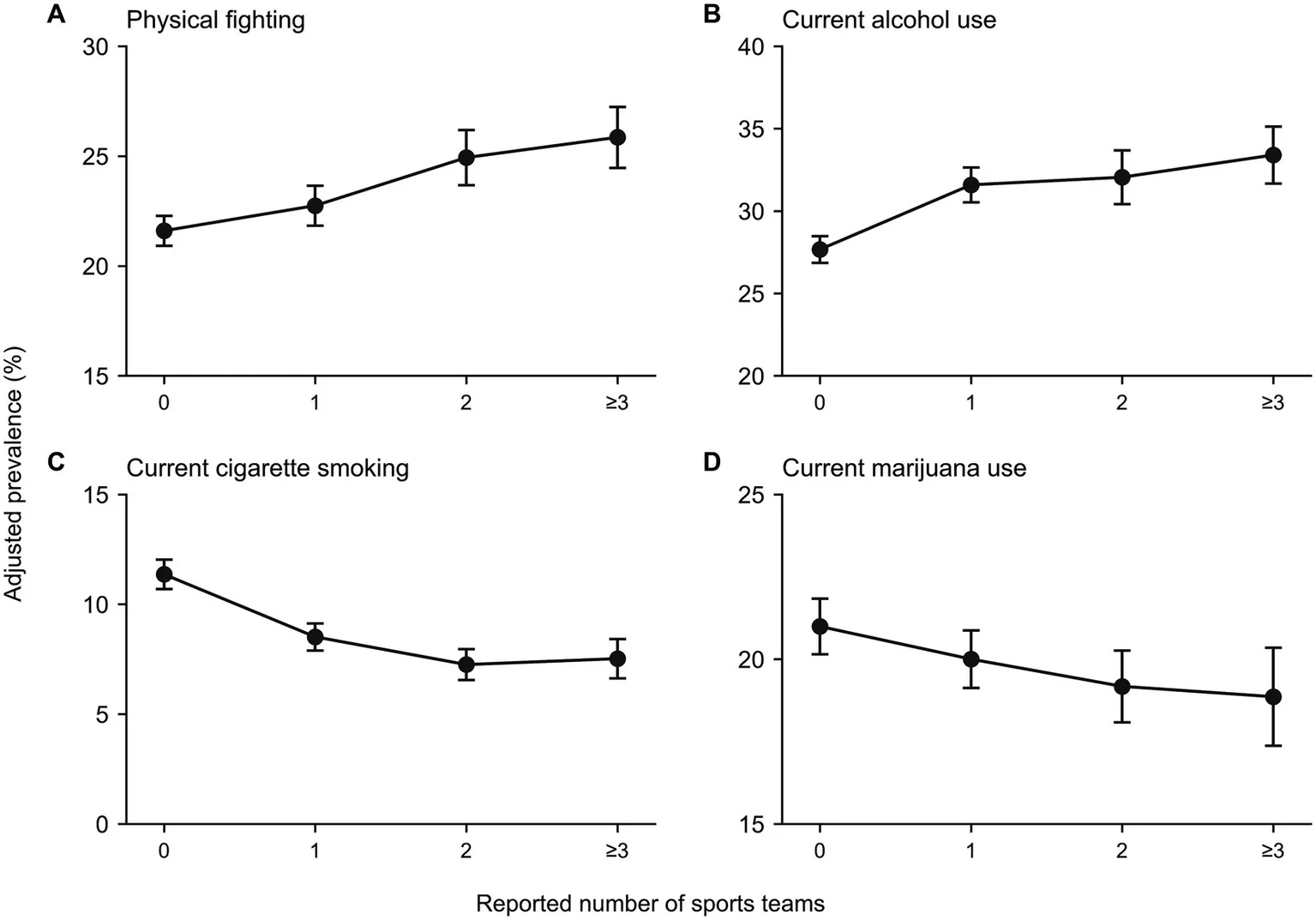
Sex-specific adjusted prevalence differences in primary risk behaviors by sports-team participation. Points show sex-specific adjusted prevalence differences (PDs; ≥1 vs. 0 teams), and horizontal error bars show design-based 95% confidence intervals. Estimates were obtained by marginal standardization from survey-weighted logistic regression models including sports-team participation, sex, and their interaction, with adjustment for grade, seven-category race/ethnicity, and survey year. Female and male estimates are distinguished by plotting symbols in addition to color. The vertical reference line denotes no prevalence difference (PD = 0). Statistical evidence of sex heterogeneity was evaluated using joint design-based tests of the participation-by-sex interaction rather than by comparing confidence-interval overlap.

For alcohol use and cigarette smoking, the association direction was shared across female and male students, but the magnitude differed. Alcohol use was positively associated with participation in both strata, with a larger absolute difference among males than females (+6.45 vs. +2.49 percentage points). Cigarette smoking was inversely associated with participation in both strata, but the absolute difference was greater among females than males (−4.53 vs. −2.36 percentage points).

Taken together, the sex-specific estimates show that the aggregate associations did not represent a single sex-neutral pattern: the positive associations with fighting and alcohol were more pronounced among males, whereas the inverse associations with cigarette smoking and marijuana use were more evident among females.

### 3.4 Survey-cycle heterogeneity, secondary outcomes, and sensitivity analyses

Survey-cycle heterogeneity was observed for current alcohol use (interaction between sports-team participation and survey year: joint *p* = 0.0095), whereas the corresponding joint tests were not supported for physical fighting (*p* = 0.115), cigarette smoking (*p* = 0.286), or marijuana use (*p* = 0.600; Table 4). Thus, variation across independently sampled survey cycles was more evident for alcohol than for the other primary outcomes.

**Table 4:** Survey-cycle heterogeneity, secondary outcomes, and sensitivity analyses.

Analysis	Outcome	Estimate or test	Result
Survey-cycle heterogeneity	Physical fighting	Participation × survey year, joint p	0.115
	Current alcohol use	Participation × survey year, joint p	0.0095
	Current cigarette smoking	Participation × survey year, joint p	0.286
	Current marijuana use	Participation × survey year, joint p	0.600
Secondary outcome	Fighting on school property	PD (95% CI); standardized PR (95% CI)	+1.34 (+0.78 to +1.89); 1.19 (1.10–1.27)
	Binge drinking, 2017–2023	PD (95% CI); standardized PR (95% CI)	+3.68 (+2.73 to +4.62); 1.39 (1.27–1.51)
Cycle-exclusion sensitivity	Four primary outcomes	Exclude 2021; exclude 2023; exclude both	Direction retained for all four outcomes
Wording sensitivity	Marijuana use, 2011–2019	PD (95% CI); standardized PR (95% CI)	−1.78 (−2.80 to −0.77); 0.92 (0.88–0.96)
Modified Poisson sensitivity	Four primary outcomes	Modified Poisson PRs	Direction consistent with standardized PRs

PD denotes adjusted prevalence difference in percentage points for participation in one or more sports teams vs. no teams. Survey-year *p* values are joint interaction tests. Binge drinking was analyzed only for 2017–2023 because of the harmonized outcome definition. Cycle-exclusion analyses evaluated whether the direction of the four primary associations changed after removing later survey cycles. Modified Poisson models were used as a relative-association sensitivity analysis.

The two secondary outcomes were directionally consistent with their related primary outcomes. Sports-team participation was associated with higher adjusted prevalence of physical fighting on school property (PD, +1.34 percentage points; 95% CI, +0.78 to +1.89; standardized PR, 1.19) and, during 2017–2023, binge drinking (PD, +3.68; 95% CI, +2.73 to +4.62; standardized PR, 1.39). The binge-drinking association also varied by sex (joint interaction *p* = 0.003), with a larger absolute difference among males, paralleling the sex pattern observed for current alcohol use.

The direction of all four primary associations was retained after excluding 2021, excluding 2023, and excluding both cycles. Restricting the marijuana analysis to 2011–2019 likewise retained the inverse association (PD, −1.78 percentage points; 95% CI, −2.80 to −0.77; standardized PR, 0.92; 95% CI, 0.88–0.96). No planned cycle-exclusion or wording sensitivity analysis reversed the direction of the primary behavior-specific pattern. The modified Poisson sensitivity models also reproduced the direction of the standardized PRs for all four primary outcomes.

## 4 Discussion

### 4.1 Principal findings and interpretive framework

The same nominal exposure—sports-team participation—corresponded to divergent behavioral correlates across the four primary outcomes. Participation was associated with higher prevalence of physical fighting and alcohol use but lower prevalence of cigarette smoking and marijuana use, while sex-stratified analyses showed that these associations were not distributed evenly between female and male students. The contribution is comparative rather than outcome-specific: four risk behaviors and their sex heterogeneity were evaluated under the same exposure definition, survey design, covariate framework, and marginal estimands. This allows the divergent associations to be interpreted as a comparative behavioral pattern rather than as findings assembled across separate studies.

This pattern is broadly consistent with systematic evidence showing that the behavioral correlates of sport differ across outcomes rather than following a uniform health-risk gradient (3, 6–8). Longitudinal evidence is similarly heterogeneous. Sports participation has been associated with faster growth in problem alcohol use (9), higher alcohol but lower marijuana initiation (10), and greater subsequent alcohol involvement alongside lower tobacco or cannabis use (11). In contrast, a more recent Norwegian cohort found little evidence that changes in sport participation were systematically accompanied by changes in alcohol use (12). Such variation suggests that developmental period, sport context, outcome definition, and broader social setting may shape the observed associations.

Two processes provide a useful framework for interpreting this heterogeneity: selection into sport and socialization within sport. Adolescents enter organized sport with different behavioral histories, social resources, and peer affiliations, while participation subsequently embeds them within peer groups, adult relationships, norms, and opportunities. Problem Behavior Theory similarly situates adolescent behavior within interacting individual and social systems rather than attributing it to a single activity (2). Within this overarching framework, the coexistence of positive associations with fighting and alcohol use and inverse associations with cigarette and marijuana use is compatible with the premise that the same adolescent may be exposed to different combinations of social expectations, opportunities, and behavioral constraints across domains. Social Cognitive Theory provides a complementary interpretation by drawing attention to how anticipated social consequences, observational learning, perceived norms, and self-regulatory processes may differ across behaviors within the same social environment (4). Thus, the present pattern does not require sports participation to operate as a uniformly protective or uniformly adverse exposure; rather, the behavioral correlates of participation may depend on which behaviors are reinforced, discouraged, modeled, or constrained within particular sporting and peer contexts. Research on youth sport has likewise emphasized selection, shared antecedents, and reciprocal developmental processes as competing explanations for observed associations (24). Candidate pathways identified in this literature include self-regulation and self-esteem or mastery, as well as peer substance use and normative beliefs; these pathways have rarely been tested directly as mediators of the sport–substance relationship (24). The present findings do not distinguish these processes. Nor do they directly test the social-cognitive processes proposed above. They do, however, indicate that whichever combination of selection and socialization is operating, it is unlikely to relate uniformly to all behavioral domains. This distinction between an overarching framework and more specific explanatory processes is also consistent with recent theoretical work emphasizing the value of integrating broad contextual frameworks with complementary motivational and psychosocial perspectives when interpreting heterogeneous adolescent behaviors (5).

### 4.2 Behavior-specific associations within organized sport contexts

The contrast between alcohol and cigarette or marijuana use illustrates why organized sport should not be treated as a unitary social exposure. Sport can simultaneously provide structured time, performance expectations, adult oversight, peer affiliation, social status, and opportunities for interaction outside training. Longitudinal and systematic evidence shows that these features can coexist with higher alcohol involvement but lower or more variable marijuana and other drug use (6, 10, 11). Associations also differ by sport type, participation intensity, sex, and club involvement, further suggesting that the social organization of participation matters (25). From a Social Cognitive Theory perspective, such divergence is plausible if the expected social rewards, sanctions, modeled behaviors, and opportunities associated with alcohol, cigarette smoking, marijuana use, and aggression differ within otherwise similar sporting environments (4). These factors were not measured in the YRBS and should therefore be regarded as candidate explanations rather than demonstrated pathways.

Alcohol is particularly informative because it was the only primary outcome in the present study to show survey-cycle heterogeneity. Earlier U. S. longitudinal work linked greater sports involvement with faster growth in problem alcohol use (9), whereas more recent Norwegian evidence found little correspondence between within-person changes in sport participation and alcohol use (12). Sport-specific longitudinal research has also shown that alcohol associations vary across particular sports and stages of development (18). The positive alcohol association observed here is therefore consistent with an established strand of the literature, but its magnitude and persistence are unlikely to be invariant across sporting and historical contexts.

The inverse cigarette result fits a different evidence base. Systematic reviews generally report lower cigarette smoking among adolescent sport participants (7, 8), while earlier National YRBS analyses have also identified lower smoked-tobacco use in some sport-participating groups (17). At the same time, tobacco-related associations are product-specific: smokeless-tobacco use can show a different pattern from cigarette smoking (7, 17). Longitudinal research further suggests that sustained organized-activity involvement and peer exposure may be relevant to smoking trajectories, with pathways differing by sex and activity type (19). The present finding should therefore be interpreted specifically as an inverse association with current cigarette smoking, rather than as evidence of uniformly lower tobacco or nicotine-related risk. The slight rebound in cigarette smoking among students reporting three or more teams is also compatible with earlier evidence that sport–substance associations need not vary linearly with participation intensity (25). This should not be interpreted as evidence of the same U-shaped relation, however, because reported team count is not equivalent to hours or intensity of sport participation.

The fighting association also requires a context-sensitive interpretation. Meta-analytic evidence has found no overall association between sports participation and juvenile delinquency (13), and longitudinal peer-context research reported no uniform association with violent delinquency (14). More sport-specific work, however, has linked serious fighting to particular contact-sport and peer-network configurations (15). Broader reviews likewise conclude that associations between sport, aggression, and violence vary with competitive context, social norms, and the organization of youth sport (26, 27). Importantly, athletic participation itself may not be the relevant social exposure in every setting: research distinguishing participation from “jock” identity found that athletic participation did not predict nonfamily violence, whereas sport-related social identity did (28).

Taken together, these findings suggest that sport type, peer networks, social identity, competitive norms, and adult regulation may be more informative than participation status alone for understanding violence-related behavior.

### 4.3 Sex heterogeneity and the social composition of sport

Sex heterogeneity was not simply a matter of stronger associations in one group. Instead, the configuration differed by behavior: the positive fighting association was concentrated among males; alcohol was positively associated in both strata but more strongly among males; cigarette smoking was inverse in both but more strongly among females; and marijuana use was inverse among females while essentially null among males. Pooled estimates therefore concealed differences not only in magnitude but, for some outcomes, in whether an association was evident.

Previous studies support sex heterogeneity but do not identify a universal female–male pattern. Among Canadian adolescents, team-sport participation was associated with binge drinking in both sexes, whereas lower cigarette and cannabis use was concentrated among girls; associations also varied with physical and social involvement in team-sport culture (16). Earlier National YRBS research likewise identified sex- and race/ethnicity-specific substance-use profiles (17). Conversely, Mays et al. found no sex difference in the longitudinal association between sports involvement and change in problem alcohol use (9), whereas Murray et al. reported sex-specific substance-use associations (18), and Metzger et al. identified different longitudinal pathways between organized activities, peers, and cigarette smoking among boys and girls (19). Evidence from other population-based studies similarly suggests that moderation is often pathway-specific rather than a general property of one demographic or behavioral characteristic. For example, Luis reported that sex moderated direct associations between school bullying and suicidal behaviors, whereas physical activity moderated indirect associations through depressive symptoms among U. S. high school students (20). In a distinct adult population, physical activity moderated the direct association between body mass index and depressive disorders but not the corresponding indirect pathway through healthcare access (21). Although these studies address different exposures and outcomes and cannot be directly generalized to sports-team participation, they reinforce the broader point that moderators may operate differently across specific behavioral pathways.

The stronger positive alcohol association among males may be interpreted in light of evidence on masculine norms and peer influence. Among U. S. high school seniors, Iwamoto and Smiler found that conformity to masculine norms was associated with alcohol use in both sexes, but the association was more pronounced among boys; among boys, risk-taking, heterosexual-display, and playboy norms, together with susceptibility to peer pressure, were directly or indirectly related to greater alcohol use (29). Athlete-specific evidence from male NCAA student-athletes further indicates that alcohol-specific masculine norms may be especially relevant: endorsement of drinking to excess was positively associated with alcohol use and alcohol-related consequences, whereas norms emphasizing control were inversely associated with these outcomes (30). Although the latter evidence comes from college athletes and cannot be directly generalized to high school students, together these findings suggest that gendered expectations and peer norms may help contextualize why the alcohol association in the present study was larger among males.

For physical fighting, team-peer environments may be relevant because norms surrounding toughness, status, and acceptable aggression can differ across sporting contexts. Prior sport-specific evidence has identified masculinity, violent social identity, and antisocial norms as potential contributors to aggression and violence (26), while adolescent research has associated jock identity, rather than athletic participation itself, with nonfamily violence, particularly among males (28). Contact-sport culture and locker-room social dynamics are therefore plausible contextual explanations, but neither sport type nor these social processes was measured in the YRBS.

The inconsistency across studies argues against treating sex itself as a sufficient explanation. Female and male students may participate in different sports, encounter different peer and competitive environments, and experience different forms of social involvement within teams. Within the integrated theoretical framework, sex is therefore better understood as a marker of heterogeneity in the social and behavioral contexts surrounding participation than as a mechanism in itself. The theoretical considerations above cannot be evaluated directly in the YRBS because the relevant sport-specific, peer-norm, and masculinity constructs were not measured. The interaction results therefore identify sex as a dimension of heterogeneity, not the process that produces it.

### 4.4 Strengths and limitations

Several features strengthen the present analysis. Seven National YRBS cycles were evaluated within a common complex-survey framework, and four primary behaviors were analyzed using the same exposure definition, covariate structure, and marginal-standardization procedure. Reporting prevalence differences alongside standardized prevalence ratios provided complementary absolute and relative perspectives. Participation-level analyses, formal sex and survey-year interaction tests, secondary outcomes, and planned sensitivity analyses further reduced reliance on a single model specification.

The principal limitations concern the level of detail available for sports participation. The YRBS sports-team measure does not distinguish individual from team sports, contact from non-contact sports, specific sport disciplines, competitive level, or season, and it does not quantify training frequency, duration, or intensity. It also lacks information on athletic identity, peer-network structure, team culture, and coaching climate. These omissions are consequential because prior research indicates that broad sport-participation measures can conceal differences by type, intensity, peer context, and activity quality (6, 14, 16, 18). Because athletic identity and social immersion were not measured, associations with team participation may also reflect correlated differences in identity or team culture that cannot be separated from participation status in these data (28). Broad dichotomous participation measures may also leave residual selection differences between participants and nonparticipants (14). Future studies that distinguish sport modality and contact characteristics and directly quantify training exposure would therefore be better positioned to identify which features of participation account for the observed behavioral heterogeneity.

The repeated cross-sectional design imposes a separate set of interpretive constraints. It prevents temporal separation of selection into sport from subsequent socialization and precludes causal inference about whether participation preceded the observed behaviors. Residual socioeconomic confounding is also possible because the harmonized analyses did not include a consistent individual-level socioeconomic measure across all seven cycles; socioeconomic conditions may influence both access to organized sport and adolescent risk behavior. Race/ethnicity was included as an adjustment variable but was not evaluated as a planned effect modifier. Prior National YRBS research suggests that sport–substance associations may vary across racial and ethnic groups (17), and the sex-specific findings should therefore not be assumed to apply uniformly across those groups. Finally, the available binary YRBS sex variable permits sex-specific estimation but cannot characterize gender identity or directly measure the gendered social processes discussed as possible contextual explanations.

Measurement error arising from self-report is another important limitation. Because sports-team participation and the behavioral outcomes were reported by the same respondents within the same survey context, common-method variance may have increased correspondence between exposure and outcome reports through shared recall processes, response tendencies, or social desirability, potentially inflating some observed associations. A related but distinct concern is differential underreporting among sports participants. Although National YRBS questionnaires are administered anonymously, students involved in organized sport may still perceive alcohol use, cigarette smoking, marijuana use, or fighting as inconsistent with team rules, athletic codes of conduct, or eligibility expectations. If such perceptions lead sports participants to underreport these behaviors more than nonparticipants, between-group associations could be biased. The present data cannot determine whether either form of reporting bias occurred or quantify its direction or magnitude, and neither survey weighting nor covariate adjustment eliminates these possibilities.

Missing exposure information provides an additional source of uncertainty. Sports-team participation was missing or unclassifiable for 12.1% of the combined sample and was more frequently unavailable in later cycles. Outcome-specific complete-case analyses may therefore have introduced differential selection. Excluding 2021, 2023, or both cycles retained the direction of all four primary associations, but these sensitivity analyses cannot identify the underlying missingness mechanism.

The substance-use outcomes also require product- and period-specific interpretation. The cigarette outcome captures conventional combustible cigarette smoking rather than the broader nicotine-product environment. A separate analysis of the 2015–2019 National YRBS found lower odds of exclusive cigarette use but higher odds of exclusive electronic-vapor-product use among sports-team participants (31), reinforcing the need to distinguish product-specific associations within adolescent nicotine use. This distinction is particularly important given the substantial change in the U. S. youth nicotine-product landscape during 2011–2023. National Youth Tobacco Survey data show that current e-cigarette use among high school students increased from 1.5% in 2011 to 13.4% in 2014, while current cigarette smoking declined from 15.8 to 9.2%; by 2014, e-cigarettes were the most commonly used tobacco product (32). In 2023, e-cigarettes remained the most commonly used tobacco product among high school students, with current use of 10.0% compared with 1.9% for cigarettes (33). The inverse cigarette association observed here should therefore be interpreted specifically as an association with combustible cigarette smoking and should not be generalized to vaping, other nicotine products, or overall nicotine exposure; nor does the contrasting pattern establish substitution from cigarettes to vaping. Marijuana measurement also changed in wording across the study period, although the planned 2011–2019 sensitivity analysis retained the inverse association.

### 4.5 Implications for youth sport and future research

The absolute differences provide useful perspective on the practical magnitude of the findings. Across the four primary outcomes, adjusted prevalence differences ranged from 1.49 to 4.46 percentage points in absolute magnitude, with alcohol showing the largest positive difference and cigarette smoking the largest inverse difference. There is no established threshold that permits these contrasts to be classified uniformly as “small” or “large.” Their importance lies instead in the coexistence of opposing associations across behaviors within a widespread adolescent activity.

The present findings identify heterogeneity in behavioral associations but do not identify the modifiable features responsible for that heterogeneity. Positive youth development research nevertheless provides a useful external framework for considering practice: constructive sport experiences have been linked to supportive adult and peer relationships and deliberately structured developmental opportunities (34). From this perspective, the practical focus is not participation alone but the social and organizational conditions under which participation occurs. For prevention science, this distinction is important: sports-team participation should not itself be treated as a universal preventive exposure or as a marker of uniformly elevated risk. Instead, school-based prevention strategies may be more informative when they identify the specific behavioral risks and social processes present within particular team environments.

For alcohol, this directs attention toward team norms and social settings surrounding participation. For violence-related behavior, team-level norms concerning aggression and the conduct modeled or tolerated within the sporting environment warrant closer assessment. These are more actionable targets for schools, coaches, and youth-sport organizations than treating all participants as uniformly low- or high-risk. Accordingly, intervention development in U. S. high school settings could prioritize assessment of team-level alcohol norms, social events surrounding participation, norms concerning aggression and toughness, and the behavior modeled or reinforced by peers and adults. Social Cognitive Theory further suggests that interventions may be strengthened by addressing expected social consequences, normative feedback, self-regulatory skills, and supportive adult modeling rather than relying solely on general health education (4). These implications remain hypotheses for intervention development, however, because the present observational analysis did not evaluate specific team processes or intervention effects.

The sex-specific findings similarly caution against one-size-fits-all assumptions. They do not justify assigning fixed behavioral profiles or interventions to female and male athletes, but they do indicate that sport type, competitive environment, team composition, and social norms should be considered when interpreting differences across participant groups. For prevention programs, the interaction findings therefore support subgroup-sensitive assessment rather than sex-based labeling: the relevant question is whether the behavioral and social conditions associated with participation differ across groups and settings.

Future research should therefore move beyond another binary sport-vs.-no-sport comparison. Longitudinal studies should measure behavior before and during participation while distinguishing individual from team sports, contact from non-contact sports, specific sport modalities, and competitive level; quantifying training frequency, duration, and intensity; and assessing peer networks, athletic identity, team climate, locker-room norms, and coaching practices. Such designs would allow selection and socialization to be evaluated as competing or reciprocal processes and would provide a stronger basis for identifying which features of youth sport are genuinely modifiable in relation to specific behavioral outcomes. Intervention studies should then test whether modifying those features changes specific outcomes, rather than assuming that increasing sports participation alone will produce a uniform behavioral benefit.

## 5 Conclusion

Across seven National YRBS cycles, sports-team participation was associated with higher prevalence of physical fighting and alcohol use but lower prevalence of cigarette smoking and marijuana use, with important differences by sex. These contrasting associations argue against using team participation as a standalone marker of adolescent behavioral health. The more relevant question is which social and organizational features of youth sport shape particular behaviors. Longitudinal research that captures those contexts is needed to clarify the processes underlying these patterns.

## Data Availability

The original contributions presented in the study are included in the article/supplementary material, further inquiries can be directed to the corresponding author.
